# The Role of Integrins in the Development and Homeostasis of the Epidermis and Skin Appendages

**Published:** 2013

**Authors:** A.L. Rippa, E.A. Vorotelyak, A.V. Vasiliev, V.V. Terskikh

**Affiliations:** N.K. Koltzov Institute of Developmental Biology, Russian Academy of Sciences; M.V. Lomonosov Moscow State University, Biological Department, Division of Cell Biology and Histology

**Keywords:** basement membrane, hair follicle, differentiation, integrins, keratinocytes, migration, morphogenesis, proliferation, stem cells

## Abstract

Integrins play a critical role in the regulation of adhesion, migration, proliferation, and differentiation of cells. Because of the variety of the functions they play in the cell, they are necessary for the formation and maintenance of tissue structure integrity. The trove of data accumulated by researchers suggests that integrins participate in the morphogenesis of the epidermis and its appendages. The development of mice with tissue-specific integrin genes knockout and determination of the genetic basis for a number of skin diseases in humans showed the significance of integrins in the biology, physiology, and morphogenesis of the epidermis and hair follicles. This review discusses the data on the role of different classes of integrin receptors in the biology of epidermal cells, as well as the development of the epidermis and hair follicles.

## INTRODUCTION

Integrins are the major class of surface receptors that attach to the extracellular matrix (EC M) and are responsible for a cell’s interaction with its environment; these receptors process external signals into intracellular ones and induce a number of regulatory cascades. Ultimately, this can lead to a variety of cellular responses. Signals that come from intracellular receptors can regulate adhesion, migration, growth, differentiation, and death of cells. Integrin dysfunction in animals causes the development of various pathologies.

**Fig. 1:**
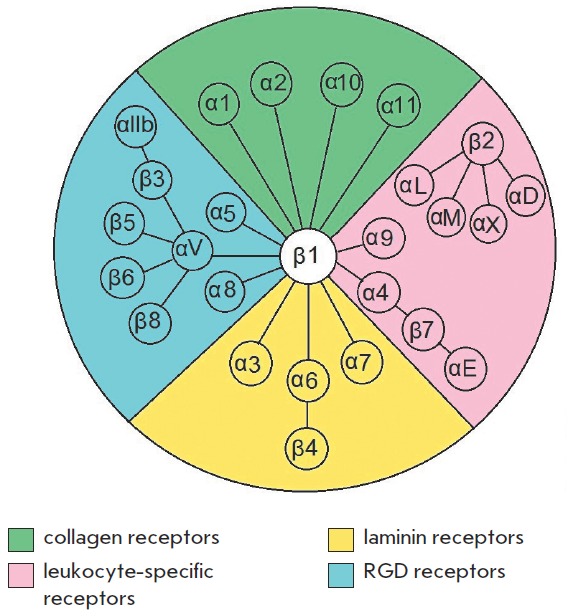
Schematic representation of the heterodimeric integrin receptors family [1, 2]

Integrins are non-covalently attached heterodimer transmembrane receptors that consist of α- and β-subunits, forming a functional receptor. Today, a total of 18 α- and 8 β-subunits are known in vertebrates. These 26 subunits form at least 24 combinations of αβ receptors (*Fig. 1*). Integrins are divided into three classes depending on the type of β-subunit. β1 integrins form the most widespread group and usually bind to EC M proteins. β2 integrins are expressed in leucocytes only; some of them can bind to the surface proteins of other cells. Some β3 integrins are expressed in thrombocytes and megakaryocytes and play a major role in the adhesion processes and blood clotting. Other β3 integrins are expressed in endothelial cells, fibroblasts, and some types of tumor cells. Receptors comprising β4-β8 subunits are rather few and have various structures; therefore, they cannot be included in any of the classes listed above [1, 2].

**Fig. 2:**
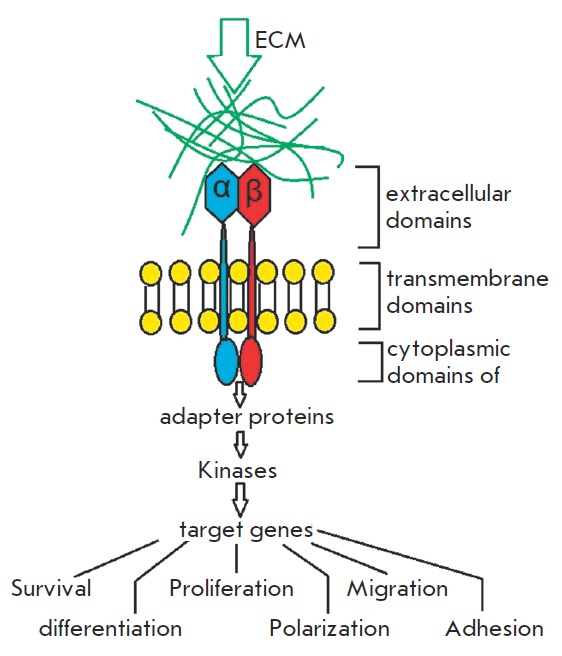
The principle of action of extracellular signals on intracellular environment processes through integrin receptors

Integrin activation on the cytoplasmic membrane from inside the cell is responsible for cytoskeletal protein synthesis and can induce the expression of some genes. On the outer side of the cell, integrins can contact with the macromolecules of EC M or basement membrane (BM) and with the receptors of other cells, thereby forming the microsurroundings of the cell. These interactions control intracellular processes and largely define the tissue structure (*Fig. 2*) [3, 4].

Integrins are responsible for the adhesion of epithelial cells to EC M by the formation of hemidesmosomes and focal adhesions.

**Fig. 3:**
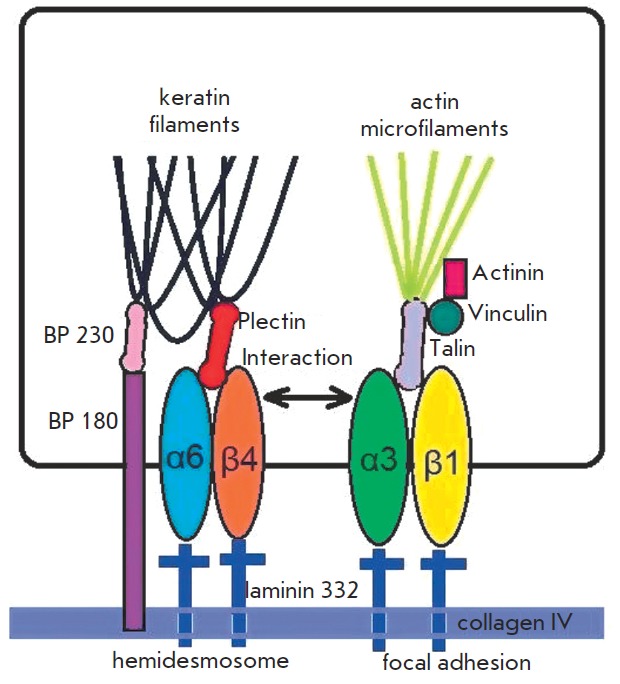
Integrin receptors in cell-matrix interactions

Hemidesmosomes are stud- and rivet-like structures on the inner side of the cytoplasmic membrane of epithelial cells. They are formed by integrin α6β4 that uses the linker proteins plectins to attach to keratin filaments and strongly immobilize epidermis on the basement membrane mostly by binding to laminin 332 (Fig. 3).

Focal adhesions are more complicated structures that are formed from integrin association and are connected to actin cytoskeleton by adapter proteins (talin, vinculin, a-actinin). The structure and morphology of these contacts are very dynamic. They can consist of hundreds of different proteins and perform adapter, signaling, and other functions. Focal adhesions are the so-called “data hubs” that regulate the protein signal flow and manage the biochemical signals of cellular responses to external stimuli (Fig. 3).

**Fig. 4:**
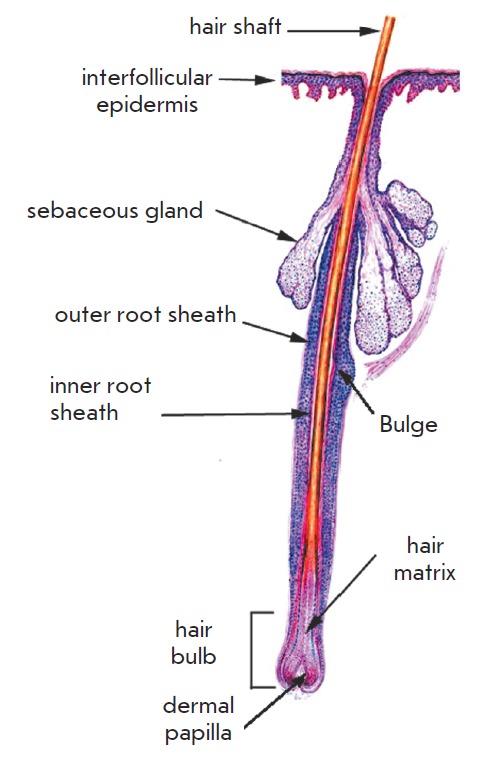
The structure of the hair follicle [36]

Integrin receptors play the key role in the formation and maintenance of the histotypical tissue structure. There is a vast amount of data supporting the role of integrins in the morphogenesis of skin epidermis and appendages, especially for the hair follicle (HF). The presence of hair is one of the defining characteristics of mammalian species. Hair has several functions, including thermoregulation, protection, sensory, and social ones. HF is developed and functions in close interaction between the epidermis and dermis. The epidermis component of HF consists of the hair matrix, the outer and inner root sheath, and the hair shaft. The dermis component of HF is represented by dermal papilla and the dermal sheath. The outer root sheath is connected to the basement layer of the epidermis from outside and to the inner root sheath from the inside; the latter surrounds the hair shaft. The outer root shaft has a thickening that is known as a bulge and contains stem cells (SC). The base of HF (bulb) is made of specialized keratinocytes of the hair matrix and mesenchymal cells of dermal papilla. The hair shaft consists of terminal, differentiated keratinocytes (trichocytes) and originates from HF. The HF is also associated with the sebaceous glands, blood vessels, nerves, and an arrector pili muscle that is attached to the bulge (Fig. 4). In the postnatal period of life, the top part of HF (including the bulge and sebaceous gland) and dermal papilla stay intact, while the other HF part undergoes changes that can be subdivided into the growth phase (anagen), transition phase (catagen), and resting phase (telogen). In mice, the anagen stage starts from the formation of HF after 14.5 days of embryonic development and continues for up to 2 weeks after birth. After that, the catagen stage occurs, which lasts for approximately 1 week and is followed by the telogen stage of approximately the same duration [5].

In humans, the anagen stage of the scalp HF lasts for 2-7 years; the telogen stage lasts for up to 3 months, and after that the hair shaft is discarded. Every HF produces on average 20-30 hair shafts during a life. Normally ~95% of all HF is in the stage of anagen, and 5% is in the telogen phase [6].

The present work reviews the role of integrins which have an important function in skin biology and integrin-linked kinase (ILK), a transmitter of intercellular integrin signaling, the functions of which have not been fully elucidated.

## EPIDERMIS INTEGRINS

There are several types of integrins that are found in the epidermis: α3β1 (predominantly a receptor for laminin 332), α6β5 (hemidesmosome component, receptor for laminin 332), and α2β1 (receptor for collagen and laminin) [7]. Integrin αvβ5 (receptor for vitronectin) is also one of the epidermal integrins, but it is expressed in lower amounts compared to other integrins [8]. In addition, basal keratinocytes of epidermis express integrins α5β1 (receptor for fibronectin) and α9β1 (receptor for tenascine C) [9, 10]. The group of β1 integrins is generally located on the basal surface of keratinocytes [7, 8, 11] and is involved in the formation of focal adhesions. Integrin α3β1 can be found on either the basal or lateral surfaces of basal keratinocytes to form intercellular contacts [12]. Under normal conditions, the expression of integrins is limited to the basal layer and outer root sheath of HF, except for integrin αvβ8 that can be found in the suprabasal layers of the eyelid skin of mice [13]. During the wound healing process and other pathological conditions, including psoriasis and tumorogenesis, integrins are expressed by suprabasal keratinocytes [14]. Ectopic expression of α2, α5, and β1 integrins in suprabasal skin layers lead to hyperproliferation, differentiation disorders, and psoriasis-like phenotype formation [15].

The creation of tissue-specific integrin knockout mice and determination of the genetic basis of several skin diseases in humans has led to an understanding of the role of integrins in the physiology and morphogenesis of epidermis. It is assumed that integrins are involved not only in the binding of keratinocytes to the basal membrane, but also in the regulation of migration, proliferation, and differentiation of epidermal cells [6, 14, 16, 17].

## INTEGRIN α6β4

Initially, integrin specific antibodies were used to determine the role of integrins in the adhesion, migration, and initiation of terminal differentiation of keratinocytes; however, these antibodies disturbed the adhesion of cultured keratinocytes to different components of EC M [6, 16]. Creation of knockout mice without some integrins or their subunits allowed one to determine their role in the adhesion of keratinocytes to BM. For example, mice with deleted α6 and β4 subunits die shortly after birth, are characterized by numerous blistering on the skin, and a stratified flat epithelium caused by the absence of hemidesmosomes [18-20]. In humans, gene mutations in the α6 or β4 subunit lead to the development of epidermolysis bullosa with stomach atresia, an autosomal disease in which skin blisters and gastrointestinal tract lesions emerge and require surgery immediately after birth [21].

Integrin α6β4 attaches to laminin 332 in EC M and to keratin filaments inside the cell. This enables coordination of the cellular response depending on the condition of laminin molecules and, therefore, makes it possible to regulate keratinocyte adhesion, migration, and proliferation. This process possibly occurs through the NF-kB or MAPK-pathway, which are initiated by β4 integrin, or through small GTPase Rac1 activation. Moreover, α6β4 integrin is required for the retention of hemidesmosome integrity. It was shown that phosphorylation of Ser1424 in the endodomain of β4 integrin leads to disintegration of the hemidesmosomes that are located on the backside of the migrating cell [22]. Integrin α6β4 can bind to collagen XVII type and plectin molecules. By using this binding and also by folding its cytoplasmic domain, integrin α6β4 may be involved in the gemidesmosome assembly [12]. It has recently been shown that silencing of the α6 subunit expression causes a significant decrease in the expression of the α3 and α2 subunits on the surface of human keratinocytes. Interestingly, this type of cells lose the capability of rapid and directed migration on laminin and type I collagen surfaces. It is assumed that integrin α6β4 can be the primary regulator of all other epidermal integrin types [23]. Meanwhile, in keratinocytes extracted from patients with β4 gene mutation, the expression level of the α3 and α6 subunits remained within the normal range [24].

The data on the role of α6β4 integrin in the cell migration process is still being debated. Possibly, this is connected to the partial interchangeability of the receptors α3β1 and α6β4 that can bind to laminin 332. According to entrenched notions, α3β1 integrin upon binding to laminin 332 provides cellular adhesion, mobility, and assembly of laminin, whereas integrin α6β4 upon binding with laminin 332 provides stable cell adhesion* via *the formation of hemidesmosomes (Fig. 3).

However, according to some data, the haptotactic migration along laminin 332 is facilitated by the combined action of both integrin receptors, and α6β4 integrin has a transdominant inhibitory effect on α3β1 (i.e., the function of α3β1 can be suppressed upon α6β4 binding). However, the use of anti-α6β4 antibodies had no effect on chemotaxis [25]. Further, it was hypothesized that the inhibition of α6β4 binding to a ligand leads to activation of an additional chemotaxic pathway, which utilizes α3β1 integrin, but cells migrate separately from each other [26]. Integrin-α6β4-deficient cells do not respond to the addition of the epidermal growth factor (EGF) either due to a lack of interaction between the EGF receptor (EGFR) and β4 or due to the suppression of integrin α3β1 activity. In the case of α6β4 expression and consequent binding upon the addition of EGF, activation of Rac1 was observed and led to the suppression and relocalization of α3β1 integrin from basal focal adhesions to the area of intracellular connections. This contributed to the migration of keratinocytes as a single layer. It is assumed that integrin α6β4 coordinates chemotaxis in the wound healing process. At wound sites, the kinetics of α6β4 integrin binding to EC M proteins changes along with the synthesis rate of its components. During the migration, integrin α6β4 binds to the secreted laminin 332. This enhances Rac1 activity and causes chemotaxis suppression (dependent on α3β1), which could be necessary to maintain communication between the leading cells and the whole layer of epithelial cells [26].

## INTEGRINS β1

In addition to α6β4 integrin, the connection to the basal membrane in the skin can be provided by integrins with a β1 subunit in their structure.

As opposed to the knockout of subunits α6 and β4, the complete knockout of β1 integrins leads to early natal destruction, which makes it impossible to define its role in the skin [27, 28].

Trying to estimate the role of β1 integrins in epidermis biology, keratinocytes were derived from a β1-null- ESC mouse. The β1-null-ESC line was obtained with a transfection vector that deactivates the β1 integrin gene [29]. β1-null-ESC expressed simple keratins *in vitro*, but they were incapable of differentiating into keratinocytes and expressing the epidermal specific keratins 14, 10, and involucrin.

It is interesting that in teratomas, which are formed after subcutaneous transplantation of β1-null-ESCs into syngeneic mice, the expression of α6β4 integrin, keratins 14, 10, and involucrin was found, attesting to the differentiation of these cells into keratinocytes.

β1-null-keratinocytes were also found in the epidermis of chimeric mice (wild type/β1-null); these mice were characterized by a normal skin. EC M proteins assembly was significantly disturbed (smaller number, thinner, and shorter BM protein filaments) in the β1- null-ESC, but it was in the normal range in the teratomas and skin of chimeric mice [30].

Since keratinocytes and dermal fibroblasts contribute to the formation of BM [31], the authors suggested that the inability of β1-null-ESC to *in vitro *differentiate into keratinocytes could be a result of the inability to produce BM proteins rather than the absence of β1 subunits alone. The observed *in vivo *differentiation into keratinocytes could happen due to the formation of BM proteins by wild type cells from the surrounding tissue [30].

However, later experiments showed that there is at least one other possible explanation for the observed phenomena. Neither contact with the BM nor the presence of normal epidermal keratinocytes does restore the ability of β1-null-ESC to differentiate into keratinocytes [32]. In the study performed on the de-epithelized “dead” dermis with a retained BM, β1-null-ESC did not differentiate into keratinocytes. Co-culturing with normal epidermal keratinocytes was not effective, either. However, introduction of normal dermal fibroblasts into the dermis led to the formation of a high number of epidermal cysts from wild-type ESC and also some keratin-14-positive cells that were differentiated from β1-null-ESC. Fibroblasts in the tissue stimulated the differentiation of keratin-14-positive cells in embryoid bodies of wild-type and β1-null cells. It was shown that the keratinocyte growth factor (KGF), fibroblast growth factor 10 (FGF10), and transforming growth factor α1 (TGFα) that were all expressed by fibroblasts would stimulate ESC to differentiate into epidermal type cells. Meanwhile, the effect of the growth factors was more obvious in the β1 knockout cells [32]. This could be explained by the fact that the concentration of growth factors in the growth medium was not the limiting factor for wild-type ESC. Therefore, for the stimulation of β1-null-ESC differentiation one needed to use a high concentration of inductors. These findings confirm the well-known synergism of integrins and growth factors and also indicate its presence at early stages of development, including the skin development process. It should also be noted that ESC of β1 gene knockout mice were unable to grow in the presence of feeder cells, while for wild-type ESC the non-proliferating fibroblasts are necessary as a feeder culture. In this regard, the question about the role of feeder cells in ESC differentiation into keratinocytes arises.

Development of technologies for the generation of mice with tissue-specific gene knockout has allowed investigators to avoid difficulties in the investigation of the mutations that lead to natal embryo death. In order to investigate the consequences of epidermis specific deletion of β1 integrins, mice with alleles flanked by the LoxP-sites of the β1 subunit gene were crossbred with mice expressing Cre-recombinase under the control of keratin 14 and the promoter of 5 genes, which are activated in the basal layer of embryonic epidermis [33, 34]. The offspring of these mice exhibited epidermal blistering, but less developed than that in the mice with subunits α6 or β4 knockout.

In the thin and fragile skin of mice with epidermisspecific β1 subunit knockout (keratin-14-promotercontrolled Cre-recombinase) there was an almost complete lack of BM, hemidesmosomes instability, a sharp decline of the proliferative potential of epidermis, and the inability of developing HF to invaginate into the dermis. These pups usually died within several hours after birth, possibly due to the lack of an epidermal barrier and dehydration. Nevertheless, the keratinocyte terminal differentiation program remained the same [33], which was contrary to the findings of some studies performed using transfection of mutant β1 subunits *in vitro *and studies of keratinocyte cultures [35,37]. The findings indicate the crucial role of integrins containing the β1 subunit in the maintenance of the proliferative capacity of the developing epidermis [33]. The inability of developing HF to invaginate into the dermis is rather interesting. The molecular mechanisms underlying the process of invagination of the growing HFs and their remodeling of EC M have not been thoroughly studied. Clearly, an important role in this process is played by integrins, particularly those containing the β1 subunit.

Mice with β1 subunit knockout in the embryo skin (Cre-recombinase controlled by the keratin 5 promoter gene) were developed. These mice were viable for 4 - 6 weeks [34], and by that time they had completely lost their HFs. Mutant mice were developing anomalies of HF and progressing hair loss due to a decrease in the proliferation of hair matrix cells. As a result, the deformed HF were replaced by macrophage infiltration; the epidermis of the back skin thickened; the basal layer of the epidermis was disorganized; cells had an abnormal morphology, irregularities in the formation of BM were observed; the number of hemidesmosomes decreased; and blistering developed. In contrast to the previous study, there was an increase in the number of layers of differentiated keratinocytes in the epidermis. The integrity of the BM surrounding the HF was not disrupted, possibly due to lower mechanical stress compared to the interfollicular epidermis or the lower structural dependence of the BM around the HF on β1 integrins. Finally, the dermal fibrosis was developed in these mice [34]. There was also a reduction in the keratinocyte proliferative potential, and some researchers suggest that this may be caused not by the lack of a β1 subunit, but by an associated decrease in integrin α6β4 expression.

The results obtained in both studies investigating the impact of the deletion of β1 integrins in embryonic epidermis attest to the important role of β1 integrins in the formation of HF, organization of BM, and proliferation and differentiation of keratinocytes. HFs are known to be degenerated and to be incapable of cyclical changes when integrin β1 is removed. Thus, it was assumed that β1 integrins are involved in the retention of the SC compartment or SC activation during the initiation of the anagen phase [34]. However, results of epidermisspecific β1 integrin gene knockout were different depending on which particular tissue-specific gene (K5 or K14) was used for Cre-recombinase activation.

Some of the mice with tissue-specific β1 integrin gene knockout were able to live for a relatively long time. This allowed one to conduct wound-healing experiments, which confirmed that β1 was required for keratinocyte migration [38].

Epidermis-specific deletion of the β1 integrin that was induced in embryogenesis [33, 34] did not allow one to fully assess its impact on proliferation, differentiation, development, and maintenance of the HF programs due to the developing fibrosis, inflammation or death of animals. To distinguish the primary effects of the β1 subunit knockout from secondary ones, researchers compared the effects of β1 integrin gene knockout in the 14.5-day mouse epidermis (using Cre- recombinase under the control of the keratin 5 gene promoter, K5Creβ1null) and induced deletions in adult epidermis (with 4-hydroxytamoxifen and CreER -recombinase under the control of the keratin 14 gene promoter, K14CreER ) [39]. In the first case (K5Creβ1null), the authors observed an increased number of differentiated cell layers, degeneration of the HF and sebaceous glands, reduced proliferation, and separation of the epidermis from the underlying derma. These animals were found to have abnormal collagen type IV accumulation and laminin 332 in the derma. The removal of β1 integrin subunits in the embryonic epidermis (K5Creβ1null) caused a disruption in terminal differentiation, which led to an increased number of cell layers expressing the markers typical of differentiated keratinocytes (keratin 10, kornifin, lorikrin and transglutaminase 1). These findings are in disagreement with data indicating the retention of the epidermal cell differentiation program in mice with tissue-specific β1 integrin knockout [33]. The remaining HF still expressed SC markers at a high level. In the second case (K14CreER ), knockout of β1 genes in the adult animal epidermis led to minor changes in the epidermis. The main abnormality observed was an increase in the number of melanocytes. Disturbance of interfollicular epidermis differentiation and reduced size of the sebaceous glands were also observed. HF remained, but the outer root sheaths of HF were increased, some HF bulbs were thin and elongated, and a significant number of proliferating cells were found in some areas of interfollicular epidermis. The high expression of SC markers in the bulge area remained on day 30 after treatment with hydroxytamoxifen.

The phenotypic changes observed after the removal of β1 integrins in mature epidermis were much less pronounced than those that occurred after the deletion of genes during fetal development. In both cases, no obvious changes in the HFSC compartment were observed [39].

Since the described animal models with different β1 subunit expression defects did not take into account the contribution of specific integrins α-subunits to regulation, the effect of α3β1 integrin lacking on mature skin and the development of the HF was studied [40].

α3β1 integrin is abundantly expressed in the skin; it localizes between hemidesmosomes and connects the BM to the actin cytoskeleton *in vivo*. Inactivation of the α3-integrin subunit resulted in the death of pups shortly after birth; kidney and lung defects were observed in the animals [41]. Pups of α3null-mice developed blisters on footpads, while the structure of hemidesmosomes remained normal. An analysis of the laminin 332 expression showed disorganization of the BM zone. The program of epidermis differentiation and stratification was unchanged [42]. Subsequent experiments showed that integrins α3β1 and α6β4 were not important for morphogenesis and homeostasis in the epidermis of the developing skin if the epidermis remained attached to the dermis. Mouse embryos lacking these integrins had a normal proliferation program and apoptosis rate in the intact BM areas until blister formation [43]. It was the contact between the epidermis and dermis, which was ensured by an unknown compensatory mechanism for a short period, which was important. With the development of blisters during embryogenesis, the intensity of apoptosis increased. Unfortunately, the morphogenesis of HF upon removal of integrins α3β1 or α6β4 has not been discussed [43].

To further investigate the consequences of integrin α3β1 removal, the skin of newborn knockout animals was implanted in nude mice. In mature grafts, disruption of BM organization in interfollicular epidermis was observed and severe morphological abnormalities of HF occurred after the first development cycle: HF growth retardation, disorganization of F-actin in HF, fragmentation of HF, variation in pigment accumulation, and the formation of HF clusters. A closer look at the transplants led to the conclusion that α3β1 integrin was not required for the differentiation of a mature interfollicular epidermis but was necessary for the regulation of various processes of morphogenesis and maintenance in HF. With α3β1 integrin deletion, a mature skin can fully develop and form the HF and sebaceous glands, therefore suggesting that α3β1 integrin is not required to maintain epidermal SC. However, significant disturbances emerged in HF after the first cycle; proliferation and apoptosis decreased, thus indicating a longer resting phase of the HF, while the formed clusters may result from unsuccessful attempts by HF to start the next growth phase. These data show that α3β1 integrin plays an important role in the specific regulation of the morphology of HF during the catagen phase of the HF cycle [40].

Taking into account the data on the role of β1 integrins in the formation and maintenance of HF, proliferation and differentiation of keratinocytes, structuring of BM, and possible involvement in the maintenance or activation of the SC population, one can assume that at least the expression of the key genes involved in the development and formation of HF was altered in mice with activated β1 integrin receptors. The phenotypic changes observed during the inactivation of these genes were similar to the phenotype that develops due to β1 integrin gene knockout.

Some transcription factors, such as hairless, complex β-catenin-LEF-1-TC F-1, or Sonic hedgehog, were found to be involved in the proliferation of hair matrix keratinocytes and HF primordia [44, 45]. The mutant phenotype leading to the inactivation of these proteins partially overlaps with the β1-null-HF phenotype. On day 15 after birth, mice with a hairless mutation had premature apoptosis and increased proliferation rate of hair bulb matrix keratinocytes; improper location of the inner root sheath and outer root sheath atrophy was observed. The outer root sheath and hair bulb were broken down into separate cell clusters [44]. Mice with gene LEF-1 inactivation lacked whiskers and HF, as was observed in mice with epidermis-specific β1 integrin gene knockout [46]. Mouse skin grafts of Shh-/-, which were implanted into immunodeficient animals, could correctly differentiate to form hyperproliferative follicle-like structures that are incapable of producing mature hair shafts [47, 48]. It is interesting to see whether the keratinocyte-specific mutations leading to enhanced activity of these proteins are able to at least partially restore the β1-null-HF phenotype.

## INTEGRIN-LINKED KINASE

Binding to a ligand induces integrin clustering, giving rise to complexes consisting of a large number of molecules. The affinity of integrin ligands is regulated by intracellular signals, thus activating integrins. The key activation regulators are talins and kindlins, which bind to the β1 and β2 integrin cytoplasmic domains [49]. The intracellular signaling pathway upon binding of integrins to EC M proteins has not been fully studied. Integrins lack either enzymatic activity or actin binding sites. It is assumed that signals are transmitted by various kinases and protein mediators.

It is most likely that integrin binding to the actin cytoskeleton is mediated by talin, α- actinin, and vinculin. Talin is required for stress transmission to the substrate via the formation of adhesive contacts, binding of integrin to the cytoskeleton, and subsequent cell flattening [50]. Talin can bind integrins to actin via different ways: directly and through vinculin, which in turn binds to α-actinin and actin. Removal of α-actinin also inhibits the formation of adhesive contacts, but its role in the force transmission to the substrate has not been studied yet [51]. Vinculin gene knockout, contrary to the talin and α-actinin genes, has no dramatic consequences. Apparently, vinculin is important for adhesion strength but is not critical for their formation [52].

Integrin-mediated contacts are very complex structures which can include over 150 different molecules [53, 54]. These complexes comprise integral membrane proteins (integrins, syndecans), actin binding proteins (talin, vinculin , α-actinin), and signaling and adapter proteins (Src tyrosine kinase, focal adhesion kinase (FAK), paxillin and ILK) [55-60]. Focal adhesions also contain p21-activated kinase (PAK), Rho GTPases, which regulate the actin polymerization, myosin 2 contraction, microtubules dynamics and organization [61], calcium-dependent calpain 2 protease [62] and tyrosine phosphatase SHP-2 [63] , which are likely to temporarily bind to adapter proteins and regulate their migration.

Protein kinase ILK, another component of focal adhesions [59], was originally identified as a protein interacting with β1 integrins [64]. ILK is required for survival, migration, and cell adhesion. It mediates the interactions with various proteins, including β1 and β3 integrins, PINC H, paxillin and parvins, thus acting as a mediator between integrins and the actin cytoskeleton [59, 65].

ILK kinase activity and phosphorylation of some proteins, including protein kinase B (PKB/Akt) and glycogen synthase kinase 3β (GSK 3β), have been described in several papers [71, 73].

GSK 3β was found in the bulge area of mature human HF tissue cultures, where it co-localizes with bulge markers, such as cytokeratin 15, 19, and CD200. Inhibition of glycogen synthase activity in this region increases the proliferation rate of outer root sheath cells, suggesting a possible involvement of GSK 3β in maintaining the SC compartment of HF [66]. The development and cyclic changes in HF in a postnatal organism substantially depend on GSK 3β inactivation [67, 68]. Active and unphosphorylated GSK 3β can bind and phosphorylate β-catenin with the APC protein, resulting in the degradation of β-catenin. Phosphorylation of GSK 3β inactivates the kinase and leads to the stabilization and translocation of β-catenin into the nucleus, where it interacts with the DNA-binding Lef1/Tcf proteins, which activate the transcription of target genes, such as the cyclin D genes, homeobox-containing transcription factors c-myc, Lef 1, and hair keratins [69, 70]. ILK, by phosphorylating GSK 3β [71, 72] or inhibiting the β-catenin degradation complex [73], can modulate β-catenin stability and thus play an important role in HF morphogenesis.

Nevertheless, the functions of ILK have not been completely elucidated, since both *in vitro *and *in vivo* findings indicate that ILK exhibits an adapter rather than kinase activity [74-80]. It is assumed that ILK contains a pseudo-kinase site that cannot be phosphorylated [81]. Thus, the degree of GSK 3β and PKB/ Akt phosphorylation in fibroblasts lacking ILK was the same as that in the control. Apparently, ILK is not involved in the phosphorylation of these kinases [74].

This hypothesis is confirmed by additional data showing that ILK regulates neither the phosphorylation of GSK 3β, nor stability or activity of β-catenin in the HF, nor the cell differentiation matrix to the inner root sheath and the hair shaft. Keratinocyte-specific ILK (K5-Cre) gene knockout in mouse (keratin 5 gene promoter controlled Cre-recombinase) led to the disturbance of keratinocyte adhesion and BM integrity, blisters formation, keratinocyte ectopic proliferation in the suprabasal layers, abnormal keratinocyte differentiation, epidermal hyperplasia, defects in HF formation, and alopecia. The disruption of HF formation is associated with the accumulation of proliferating cells in the outer root sheath; while cell differentiation in the HF matrix and maintenance of SC remained the same. Mice with ILK gene knockout lived for a long time [80].

In contrast to the knockout of β1-integrin genes, which reduces the proliferation of epidermal keratinocytes and HF matrix cells [33, 34], the knockout of ILK (K5-Cre) led to an insignificant decrease in the number of proliferating cells in the HF matrix. On the contrary, a substantial increase in the number of proliferating cells was observed in the outer root sheath. Since outer root sheath cells originate from the CD34^+^- stem cell population in the bulge [82], the authors checked whether the absence of ILK affects this population of cells. HF was found to contain CD34^+^-cells that will differentiate into transient cells. Since proliferating cells were accumulated in the outer root sheath but not in the hair matrix, a conclusion was made that ILK was required so that transient cells could migrate into the matrix and a hair bud could form during the anagen phase.

Interestingly, the absence of ILK in the keratinocyte culture influenced the formation of focal adhesions and prevented sustained directional migration. Cells also exhibited weak integrin mediated adhesions, and therefore did not capture lamellipodia, leading to changes in migration [80].

The consequences of ILK removal induced by cDNA expression in Cre-recombinase under the control of the keratin 14 gene promoter (K14-Cre) were also studied. In contrast to K5-Cre tissue-specific knockout [80], mice survived on average for up to 4 days after birth when this method was used for ILK gene inactivation. It should be noted that with β1 integrin gene knockout, when the expression of Cre-recombinase was controlled by the keratin 14 gene promoter, the animals died soon after birth; while surviving for up to 6 weeks when the keratin 5 gene promoter was used [33, 34]. These differences can be explained if one takes into account the fact that keratin 14 expression starts after 11.5 days of embryonic development [83], and keratin 5 expression starts after 15 days [80], when the epidermis is already stratified and HF morphogenesis has begun. Such phenotypic differences may reflect the manifestation of the activity of keratin genes or differences in the intensity and/or expression time of the Cre-transgene and the ILK gene inactivation during embryogenesis.

Hence, deletion of ILK using the K14-CRE system weakened the morphogenesis of HF. Since the HF proliferation decreased, the number of HF decreased and morphogenesis could not be fully completed. The absence of ILK caused abnormalities in hemidesmosomes and triggered multiple formation of micro-blisters, dearrangement of keratinocytes in the suprabasal layers and the actin cytoskeleton, impaired adhesion, polarization, and migration.

ILK is considered to be a β1-integrin target. The absence of ILK and β1-integrins in the skin leads to a number of similar disturbances, including abnormal formation and performance of HF, a decrease in the proliferative activity of follicular keratinocytes, and blisters development.

Keratinocytes lacking ILK developed defects in adhesion and proliferation *in vitro*. The reduced proliferation rate resembled the disorders observed in HF but not those in interfollicular epidermis. The normal proliferation of primary keratinocytes in a cell culture is known to depend on the activation of α3β1-integrins [84]. Taking in account the fact that the primary keratinocyte culture consists of transient and committed progenitor cells, the reduction in the proliferation rate of keratinocytes lacking ILK could be a result of intracellular β1-integrin signaling disruption in this cell population. Possible disorders in SC proliferation could also be the cause, but this hypothesis is yet to be verified.

Upon ILK gene inactivation, adhesion and proliferation disturbances, as well as polarization and migration of murine keratinocytes in the cell culture, were observed [85]. The key event in polarization is the activation of Rac1 on the leading edge of the cell, causing the formation and stabilization of lamellipodia with integrin α3β1 [86]. Infection of cells with an adenovirus carrying a constitutively active Rac1 reduces the polarization defects in ILK-deficient keratinocytes. Thus, ILK is a crucial component of the signaling pathway, which connects integrin stimulation with Rac1 recruitment to the membrane, with spreading activation and directed migration of keratinocytes [85]. Lamellipodia stabilization defects could also be observed if the normal cells were transfected with the mutant Rac1 gene. Rac1 alone is not sufficient to stabilize lamellipodia, since the Rac1 constitutive expression in integrin- α3β1-deficient keratinocytes did not restore the type of migration [86].

The consequences of ILK inactivation in the HFSC were studied using tissue-specific knockout of the K15- Cre system that is specific of the HFSC [87].

During the induced inactivation of ILK in HFSC, hair follicles were able to enter the anagen phase. Stem cells from the bulge lacking ILK successfully migrated from the bulge and differentiated into cells of the outer root sheath and HF matrix and entered the growth phase. Consequently, the absence of ILK in the HFSC affects neither their migration nor their ability to produce a population of transient cells for the regeneration of HF. Meanwhile, the *in vitro *behavior of keratinocytes isolated from the bulge area of test mice (K15-Cre) differed from that of the controls. The adhesion efficiency to EC M-coated plastic was low. These findings coincide with the data obtained from keratinocytes isolated from the neonatal mouse epidermis, in which ILK was removed using K14-Cre [85]. The absence of ILK in HFSC mainly manifested itself as a reduction in the ability of bulge cells to differentiate into interfollicular epidermis cells during wound healing. Closure of the wounds on the back of experimental animals occurred later than that in the control group. The few bulge keratinocytes, which participated in the epidermis regeneration, were characterized by a low proliferative potential. Consequently, the ILK is required for the migration of bulge stem cell progeny into regenerating epidermis and for proliferation during wound re-epithelization. Taking into account the adhesion and migration defects of the keratinocytes derived from neonatal mice with epidermis- specific ILK deletion [80, 85], as well as the data on ILK inactivation in bulge SCs [87], one can assume that ILK mediates the interaction between cells and EC M, and that it contributes to the immobilization of keratinocytes on the basal membrane.

The molecular pathways modulated by ILK remain insufficiently studied. To fill in the gaps in our understanding of the role of ILK in the epidermis, some researchers have tried to determine gene expression by a microarray analysis [88]. For this purpose, gene expression in normal murine epidermis on day 3 after birth was compared to that in the epidermis with an inactivated ILK gene using tissue-specific knockout (K14- Cre). It was found that 27% of the transcripts were expressed at a lower level. These transcripts encoded hair-specific keratins and proteins associated with them, such as keratin 31, the keratin-associated protein 3-3 and others, which is consistent with the disruption observed in the HF. The expression levels of desmoglein 4 (a protein important for the structural integrity of cuticle desmosomes and the HF cortex) and trichohyalin (a component of the inner root sheath) were 18- and 28-fold lower than the normal ones, respectively. A significant decrease in the expression level of these genes is consistent with the notion of the ILK expression importance after stages 4-5 of follicle formation.

ILK also plays an important modulating role in epidermal keratinocyte differentiation and formation of the epidermal barrier. This explains the lower expression of the genes encoding the key enzymes and factors which are required for protein crosslinking and lipid biosynthesis (e.g., transglutaminase 3, the substrate for transglutaminases Prr9, and others) observed in mice with epidermis ILK inactivation.

In the absence of ILK in the epidermis, expression of the genes involved in the Wnt and Shh signaling pathways was higher. Under normal skin morphogenesis, these signaling pathways are active at the early stages of HF development, while their activity decreases at later stages. Suspension of HF development at stages 2-4 in postnatal epidermis with the absence of ILK may be an indication of an increased expression of Wnt and Shh signaling pathway genes.

A transcriptome analysis of postnatal epidermis with ILK gene knockout revealed its role in HF development, keratinocyte maturation, and formation of the barrier function, as well as in pigmentation and regenerative processes [88].

## INTEGRINS β1 AS MARKERS OF EPIDERMAL SCs

The behavior of SCs is controlled by the interaction between the internal transcriptional programs and external signals [89]. External signals are provided by the local microenvironment or niche where stem cells are located. EC M is an important component of the stem cell niche [90-93].

In the bulge region where HFSC are located, the EC M composition significantly differs from the composition of the remaining epidermis portions [94, 95]. Several-fold overexpression of collagen types VI, XVIII, V, tenascin C, periostin, cysteine-rich glycoprotein nephronectin and other EC M components are observed in this region. The functional significance of these differences remains poorly investigated. Direct involvement of EC M and integrin receptors in the regulation of the fate of epidermal SC is doubtless. Interestingly, the composition of the EC M in the central portion of the cornea, which contains differentiating cells, and in the limb that contains corneal SC also differs considerably. The limb area is enriched in collagen VII, XVI and IV, tenascin C, vitronectin, and laminin [96]. Hence, the integrin types that are expressed in these areas of cornea vary as well. Slowly proliferating and retaining the DNA label, limb cells are characterized by an overexpression of β1, β4, and α6 integrins. Small clonogenic cells of corneal rings were extracted on the basis of the α6^bright^/CD71^dim^ phenotype [97], which was also used to isolate the population of epidermal SC [98].

It has recently been shown that integrins can be used to enrich a SC population derived from various tissues [99-102].

In a human keratinocyte culture, the population of SC and transiently amplified cells were separated according to the β1-integrin expression level and the rate of adhesion to EC M proteins. The SC population with high levels of β1-integrin expression had a high colony forming efficiency and adhered to EC M proteins much faster than the cells of the transient compartment, which underwent terminal differentiation after one or five division cycles [103]. Cell motility depends on integrin expression levels, with motility being inhibited at high expression levels, and the medium level is the most favorable for cell motility [104]. Thus, transient cells that weakly express β1-integrin should have considerably higher motility than the strongly expressing SC, which was confirmed using time-lapse shooting. Moreover, transient cells were dispersed in a highdensity cell culture, as opposed to SCs, which were arranged compactly [11].

High expression of β1-integrin (bright fluorescence after antibody staining) was used as a marker to determine the spatial organization of SCs and their progeny in human epidermis [11]. Keratinocytes with a low expression of these integrins originated from the SC compartment and started to rapidly proliferate and undergo differentiation.

In mouse epidermis, β1-integrins are expressed intensively in the bulge zone of HF and are widely used as markers for this region [68]. However, the use of the β1-integrin expression for human HF bulge cells is not possible, since they are expressed throughout the external layer of the outer root sheath, connective tissue sheath, and in the dermal papilla [105]. ;

Similar results were obtained during the evaluation of the β1-integrin expression in tissue cultures of human scalp HF. The co-expression of fibronectin and tenascin C was also observed at the β1-integrin immunoreactivity sites. Researchers found no significant increase in β1-integrin immunoreactivity *in situ *in the bulge area. The use of β1-integrin activating antibodies and RGD tripeptides (Arg-Gly-Asp), which simulate natural ligands, contributed to the growth of the HF tissue cultures extracted by *in vitro *microdissection, and it prevented their spontaneous regression. Thus, despite the lack of β1-integrin overexpression in human HFSCs, their signaling pathways play a role in the control of follicle growth. This approach may become a potential tool for preventing hair loss in humans via direct stimulation of the intracellular β1-integrin signaling pathway [106].

It was shown that β1 integrins and MAP-kinase contribute to the *in vitro *maintenance of the SC compartment. Transfection of a human keratinocyte culture with a retrovirus containing a mutant integrin β1 subunit (dominant negative mutation) decreased the surface expression level of these subunits, cell adhesion level, and MAP-kinase activation. This resulted in the differentiation of SCs [17].

A skin chemical carcinogenesis model was used to show that epidermis-specific α3-integrin gene knockout slows the initiation step under the action of 7,12-dimethylbenz(α)anthracene and facilitates the exit of HFSC from the niche and their differentiation, thus preventing the accumulation of the transformed cells in the skin. Further treatment with phorbol ester caused no tumor progression in the experimental animals. Meanwhile, under prolonged exposure to DMBA alone in a single component protocol, tumor progression with transition to the malignant form was more effective and occurred at a higher rate in the epidermis of animals with α integrin gene knockout, although the number of malignancy lesions was lower [107].

β1 integrins are required for apical localization of the protein complexes that regulate the asymmetric division of epidermal SCs, which ensures balance between the stem and progenitor cells localized on the BM and their differentiating progeny in the suprabasal layers of the epidermis [108].

Integrins can directly activate growth factor receptors in the absence of these factors [109].

Integrin receptors combine the functions of mechanical attachment of cells to the substrate and bidirectional signaling. On one hand, they provide an adequate cellular response to the signals from the environment; on the other hand, they allow the cell to modulate its microenvironment by itself. The adhesion of basal cells to the BM in the epidermis is critical for a firm connection between the epidermis and dermis, for maintaining its histotypical epidermal structure, and performance of its protective functions. However, integrins have other functions as well. In addition to participating in the assembly of BM proteins, integrins monitor the orientation of the mitotic spindle and the apical localization of the protein complex during the asymmetric division of basal keratinocytes, contributing to the continuous regeneration of the epidermis and maintaining a pool of basal keratinocytes. Integrins adjust the migration, proliferation, and differentiation of epidermal cells, thus eventually determining the morphogenesis of the skin and its appendages. Abnormal integrin expression results in a delay in HF development during embryogenesis or in degradation and loss of hair follicles in adulthood. Abnormalities in the integrin expression may be the underlying reason of a number of pathological conditions, including malignization.

## Abbreviations

BM: basement membrane
ECM: extracellular matrix
HF: hair follicle
SCs: stem cells;
ESCs: embryonic stem cells
ILK: integrin-linked kinase

## References

[1] Hynes R.O. (2002). Cell..

[2] Barczyk M., Carracedo S., Gullberg D. (2010). Cell Tissue Res..

[3] Bouvard B., Brakebusch C., Gustafsson E., Aszódi A., Bengtsson T., Berna A., Fässler R. (2001). Circ. Res..

[4] Bokel C., Brown N.H. (2002). Dev. Cell..

[5] Stenn K.S., Paus R. (2001). Physiol. Rev..

[6] Gray J., Dawber R. A. (1999). Pocketbook of hair and scalp disorders: an illustrated guide.. Blackwell Science.

[7] Adams J.C., Watt F.M. (1991). J. Cell Biol..

[8] Hertle M., Adams J., Watt F.M. (1991). Development..

[9] Adams J.C., Watt F.M. (1990). Cell..

[10] Palmer E.L., Ruegg C., Ferrando R., Pytela R., Sheppard D. (1993). J. Cell Biol..

[11] Jensen U.B., Lowell S., Watt F.M. (1999). Development..

[12] Hashmi S., Marinkovich M.P. (2011). Clin. Dermatol..

[13] Stepp M.A. (1999). Dev. Dyn..

[14] Watt F.M. (2002). EMBO J..

[15] Carroll J.M., Romero M.R., Watt F.M. (1995). Cell..

[16] Watt F.M., Kubler M.D., Hotchin N.A., Nicholson L.J., Adams J.C. (1993). J. Cell Sci..

[17] Zhu A.J., Haase I., Watt F.M. (1999). Proc. Natl. Acad. Sci. USA..

[18] Dowling J., Yu Q.C., Fuchs E. (1996). J. Cell Biol..

[19] Georges-Labouesse E., Messaddeq N., Yehia G., Cadalbert L., Dierich A., LeMeur M. (1996). Nat. Genet..

[20] Van der Neut R., Krimpenfort P., Calafat J., Niessen C.M., Sonnenberg A. (1996). Nat. Genet..

[21] Ashton G.H., Sorelli P., Mellerio J.E., Keane F.M., Eady R.A., McGrath J.A. (2001). Br. J. Dermatol..

[22] Germain E.C., Santos T.M., Rabinovitz I. (2009). Mol. Biol. Cell..

[23] Kligys K.R., Wu Y., Hopkinson S.B., Kaur S., Platanias L.S., Jones J.C. (2012). J. Biol. Chem..

[24] Sehgal B.U., DeBiase P.J., Matzno S., Chew T.L., Claiborne J.N., Hopkinson S.B., Russell A., Marinkovich M.P., Jones J.C. (2006). J. Biol. Chem..

[25] Hintermann E., Bilban M., Sharabi A., Quaranta V. (2001). J. Cell Biol..

[26] Russell A.J., Fincher E.F., Millman L., Smith R., Vela V., Waterman E.A., Dey C.N., Guide S., Weaver V.M., Marinkovich M.P. (2003). J. Cell Sci..

[27] Fassler R., Meyer M. (1995). Genes Dev..

[28] Stephens L.E., Sutherland A.E., Klimanskaya I.V., Andrieux A., Meneses J., Pedersen R.A., Damsky C.H. (1995). Genes Dev..

[29] Fässler R., Pfaff M., Murphy J., Noegel A.A., Johansson S., Timpl R., Albrecht R. (1995). J. Cell Biol..

[30] Bagutti C., Wobus A.M., Fassler R., Watt F.M. (1996). Developmental Biology.

[31] Marinkovich M.P., Keene D.R., Rimberg C.S., Burgeson R.E. (1993). Dev. Dyn..

[32] Bagutti C., Hutter C., Chiquet-Ehrismann R., Fässler R., Watt F.M. (2001). Developmental Biology.

[33] Raghavan S., Bauer C., Mundschau G., Li Q., Fuchs E. (2000). J. Cell Biol..

[34] Brakebusch C., Grose R., Quondamatteo F., Ramirez A., Jorcano J.L., Pirro A., Svensson M., Herken R., Sasaki T., Timpl R. (2000). EMBO J..

[35] Levy L., Broad S., Diekmann D., Evans R.D., Watt F.M. (2000). Mol. Biol. Cell..

[36] www.vetmed.vt.edu./education/curriculum/vm8054/Labs/Lab15/Lab15.htm.

[37] Hotchin N.A., Gandarillas A., Watt F.M. (1995). J. Cell Biol..

[38] Grose R., Hutter C., Bloch W., Thorey I., Watt F.M., Fässler R., Brakebusch C., Werner S. (2002). Development..

[39] Lopez-Rovira T., Silva-Vargas V., Watt F.M. (2005). J. Invest. Dermatol..

[40] Conti F.J., Rudling R.J., Robson A., Hodivala-Dilke K.M. (2003). J. Cell Sci..

[41] Kreidberg J.A., Donovan M.J., Goldstein S.L., Rennke H., Shepherd K., Jones R.C., Jaenisch R. (1996). Development..

[42] DiPersio C.M., Hodivala-Dilke K.M., Jaenisch R., Kreidberg J.A., Hynes R.O. (1997). J. Cell Biol..

[43] DiPersio C.M., van der Neut R., Georges-Labouesse E., Kreidberg J.A., Sonnenberg A., Hynes R.O. (2000). J. Cell Sci..

[44] Panteleyev A.A., Botchkareva N.V., Sundberg J.P., Christiano A.M., Paus R. (1999). Am. J. Pathol..

[45] Oro A.E., Scott M.P. (1998). Cell..

[46] Van Genderen C., Okamura R.M., Farinas I., Quo R.G., Parslow T.G., Bruhn L., Grosschedl R. (1994). Genes Dev..

[47] St-Jacques B., Dassule H.R., Karavanova I., Botchkarev V.A., Li J., Danielian P.S., McMahon J.A., Lewis P.M., Paus R., McMahon A.P. (1998). Curr. Biol..

[48] Chiang C., Swan R.Z., Grachtchouk M., Bolinger M., Litingtung Y., Robertson E.K., Cooper M.K., Gaffield W., Westphal H., Beachy P.A. (1999). Developmental Biology.

[49] Shattil S.J., Kim C., Ginsberg M.H. (2010). Nat. Rev. Mol. Cell. Biol..

[50] Zhang X., Jiang G., Cai Y., Monkley S.J., Critchley D.R., Sheetz M.P. (2008). Nat. Cell. Biol..

[51] Choi C.K., Vicente-Manzanares M., Zareno J., Whitmore L.A., Mogilner A., Horwitz A.R. (2008). Nat. Cell Biol..

[52] Xu W., Baribault H., Adamson E.D. (1998). Development..

[53] Geiger B., Spatz J.P., Bershadsky A.D. (2009). Nat. Rev. Mol. Cell Biol..

[54] Geiger B., Yamada K.M. (2011). Cold Spring Harb. Perspect Biol..

[55] Turner C.E. (2000). Nat. Cell Biol. 2000. V. 2. № 12. P. E231–E236..

[56] Zamir E., Geiger B. (2001). J. Cell Sci..

[57] Frame M.C. (2004). J. Cell Sci..

[58] Mitra S.K., Hanson D.A., Schlaepfer D.D. (2005). Nat. Rev. Mol. Cell Biol..

[59] Legate K.R., Montanez E., Kudlacek O., Fassler R. (2006). Nat. Rev. Mol. Cell Biol..

[60] Huttenlocher A., Horwitz A.R. (2011). Cold Spring Harb. Perspect Biol..

[61] Ridley A.J. (1994). Bioessays..

[62] Beckerle M.C., Burridge K., DeMartino G.N., Croall D.E. (1987). Cell..

[63] Yu D.H., Qu C.K., Henegariu O., Lu X., Feng G.S. (1998). J. Biol. Chem..

[64] Hannigan G.E., Leung-Hagesteijn C., Fitz-Gibbon L., Coppolino M.G., Radeva G., Filmus J., Bell J.C., Dedhar S. (1996). Nature.

[65] Grashoff C., Thievessen I., Lorenz K., Ussar S., Fässler R. (2004). Curr. Opin. Cell Biol..

[66] Yamauchi K., Kurosaka A. (2010). Arch. Dermatol. Res..

[67] Fuchs E., Merrill B.J., Jamora C., DasGupta R. (2001). Dev. Cell..

[68] Huelsken J., Vogel R., Erdmann B., Cotsarelis G., Birchmeier W. (2001). Cell..

[69] Zhou P., Byrne C., Jacobs J., Fuchs E. (1995). Genes Dev..

[70] Logan C.Y., Nusse R. (2004). Annu. Rev. Cell Dev. Biol..

[71] Delcommenne M., Tan C., Gray V., Rue L., Woodgett J., Dedhar S. (1998). Proc. Natl. Acad. Sci. USA..

[72] Novak A., Hsu S.C., Leung-Hagesteijn C., Radeva G., Papkoff J., Montesano R., Roskelley C., Grosschedl R., Dedhar S. (1998). Proc. Natl. Acad. Sci. USA..

[73] Oloumi A., Syam S., Dedhar S. (2006). Oncogene..

[74] Sakai T., Li S., Dicheva D., Grashoff C., Sakai K., Kostka G., Braun A., Pfeifer A., Yurchenco P.D., Fassler R. (2003). Genes Dev..

[75] Lynch D.K., Ellis C.A., Edwards P.A., Hiles I.D. (1999). Oncogene..

[76] Zervas C.G., Gregory S.L., Brown N.H. (2001). J. Cell Biol..

[77] Mackinnon A.C., Qadota H., Norman K.R., Moerman D.G., Williams B.D. (2002). Curr. Biol..

[78] Hill M.M., Feng J., Hemmings B.A. (2002). Curr. Biol..

[79] Grashoff C., Aszodi A., Sakai T., Hunziker E.B., Fässler R. (2003). EMBO Rep..

[80] Lorenz K., Grashoff C., Torka R., Sakai T., Langbein L., Bloch W., Aumailley M., Fassler R. (2007). J. Cell Biol..

[81] Qin J., Wu C. (2012). Curr. Opin. Cell Biol..

[82] Blanpain C., Fuchs E. (2006). Annu. Rev. Cell Dev. Biol..

[83] Dassule H.R., Lewis P., Bei M., Maas R., McMahon A.P. (2000). Development..

[84] Manohar A., Shome S.G., Lamar J., Stirling L., Iyer V., Pumiglia K., DiPersio C.M. (2004). J. Cell Sci..

[85] Nakrieko K.A., Welch I., Dupuis H., Bryce D., Pajak A., St-Arnaud R., Dedhar S., D´Souza S.J., Dagnino L. (2008). Mol. Biol. Cell..

[86] Choma D.P., Pumiglia K., DiPersio C.M. (2004). J. Cell Sci..

[87] Nakrieko K.A., Rudkouskaya A., Irvine T.S., D'Souza S.J., Dagnino L. (2011). Mol. Biol. Cell..

[88] Judah D., Rudkouskaya A., Wilson R., Carter D.E., Dagnino L. (2012). PLoS One..

[89] Watt F.M., Driskell R.R. (2010). Philos. Trans. R. Soc. Lond. B. Biol. Sci..

[90] Hall P.A., Watt F.M. (1989). Development..

[91] Scadden D.T. (2006). Nature.

[92] Spradling A., Drummond-Barbosa D., Kai T. (2001). Nature.

[93] Watt F.M., Hogan B.L. (2000). Science..

[94] Morris R.J., Liu Y., Marles L., Yang Z., Trempus C., Li S., Lin J.S., Sawicki J.A., Cotsarelis G. (2004). Nat. Biotechnol..

[95] Tumbar T., Guasch G., Greco V., Blanpain C., Lowry W.E., Rendl M., Fuchs E. (2004). Science..

[96] Ordonez P., Di Girolamo N. (2012). Stem Cells..

[97] Hayashi R., Yamoto M., Saito T., Oshima T., Okano T., Tano Y., Nishida K. (2008). Biochem. Biophys. Res. Commun..

[98] Li A., Simmons P.J., Kaur P. (1998). Proc. Natl. Acad. Sci. USA..

[99] Wagers A.J., Weissman I.L. (2006). Stem Cells..

[100] Stingl J., Eirew P., Ricketson I., Shackleton M., Vaillant F., Choi D., Li H.I., Eaves C.J. (2006). Nature.

[101] Shackleton M., Vaillant F., Simpson K.J., Stingl J., Smyth G.K., Asselin-Labat M.L., Wu L., Lindeman G.J., Visvader J.E. (2006). Nature.

[102] Watt F.M., Fujiwara H. (2011). Cold Spring Harb. Perspect Biol..

[103] Jones P.H., Watt F.M. (1993). Cell..

[104] Huttenlocher A., Sandborg R.R., Horwitz A.F. (1995). Curr. Opin. Cell Biol..

[105] Kloepper J.E., Tiede S., Brinckmann J., Reinhardt D.P., Meyer W., Faessler R., Paus R. (2008). Exp. Dermatol..

[106] Kloepper J.E., Hendrix S., Bodo E., Tiede S., Humpries M.J., Philpott M.P., Fässler R., Paus R. (2008). Exp. Cell Res..

[107] Sachs N., Secades P., van Hulst L., Kreft M., Song J.Y., Sonnenberg A. (2012). Proc. Natl. Acad. Sci. USA..

[108] Lechler T., Fuchs E. (2005). Nature.

[109] Moro L., Venturino M., Bozzo C., Silengo L., Altruda F., Beguinot L., Tarone G., Defilippi P. (1998). EMBO J..

